# De Novo Lipogenesis-Related Monounsaturated Fatty Acids in the Blood Are Associated with Cardiovascular Risk Factors in HFpEF Patients

**DOI:** 10.3390/jcm12154938

**Published:** 2023-07-27

**Authors:** Matthias Bock, Clemens von Schacky, Johannes Scherr, Elke Lorenz, Benjamin Lechner, Alexander Krannich, Rolf Wachter, André Duvinage, Frank Edelmann, Katharina Lechner

**Affiliations:** 1 Department of Cardiology, German Heart Centre Munich, Technical University of Munich, Lazarettstraße 36, 80636 Munich, Germany; 2 DZHK (German Centre for Cardiovascular Research), Partner Site Munich, Munich Heart Alliance, Munich, Germany; 3 Omegametrix, 82152 Martinsried, Germany; 4 University Center for Prevention and Sports Medicine, Balgrist University Hospital, University of Zurich, 8008 Zurich, Switzerland; 5 Department of Internal Medicine IV, Ludwig-Maximilians University, 80336 Munich, Germany; 6 Charité, Universitätsmedizin Berlin, 13353 Berlin, Germany; 7 Clinic and Policlinic for Cardiology, University Hospital Leipzig, 04103 Leipzig, Germany; 8 University Medical Center Göttingen, Department of Cardiology and Pneumology, Georg-August University, 37099 Göttingen, Germany; 9 DZHK (German Centre for Cardiovascular Research), Partner Site Göttingen, Göttingen, Germany; 10 Department of Prevention, Rehabilitation and Sports Medicine, School of Medicine, Technical University of Munich, 80992 Munich, Germany; 11 Deutsches Herzzentrum der Charité, Department of Cardiology, Angiology and Intensive Care Medicine, Campus Virchow-Klinikum, Augustenburger Platz 1, 13353 Berlin, Germany; 12 Charité, Universitätsmedizin Berlin, Corporate Member of Freie Universität Berlin and Humboldt-Universität zu Berlin, Charitéplatz 1, 10117 Berlin, Germany; 13 DZHK (German Centre for Cardiovascular Research), Partner Site Berlin, Berlin, Germany

**Keywords:** de novo lipogenesis-related fatty acids, monounsaturated fatty acids, heart failure, HFpEF, metabolic phenotype, aerobic capacity

## Abstract

De novo lipogenesis (DNL)-related monounsaturated fatty acids (MUFAs) in the blood are associated with incident heart failure (HF). This observation’s biological plausibility may be due to the potential of these MUFAs to induce proinflammatory pathways, endoplasmic reticulum stress, and insulin resistance, which are pathophysiologically relevant in HF. The associations of circulating MUFAs with cardiometabolic phenotypes in patients with heart failure with a preserved ejection fraction (HFpEF) are unknown. In this secondary analysis of the Aldosterone in Diastolic Heart Failure trial, circulating MUFAs were analysed in 404 patients using the HS-Omega-3-Index^®^ methodology. Patients were 67 ± 8 years old, 53% female, NYHA II/III (87/13%). The ejection fraction was ≥50%, E/e′ 7.1 ± 1.5, and the median NT-proBNP 158 ng/L (IQR 82-298). Associations of MUFAs with metabolic, functional, and echocardiographic patient characteristics at baseline/12 months follow-up (12 mFU) were analysed using Spearman’s correlation coefficients and linear regression analyses, using sex/age as covariates. Circulating levels of C16:1n7 and C18:1n9 were positively associated with BMI/truncal adiposity and associated traits (dysglycemia, atherogenic dyslipidemia, and biomarkers suggestive of non-alcoholic-fatty liver disease). They were furthermore inversely associated with functional capacity at baseline/12 mFU. In contrast, higher levels of C20:1n9 and C24:1n9 were associated with lower cardiometabolic risk and higher exercise capacity at baseline/12 mFU. In patients with HFpEF, circulating levels of individual MUFAs were differentially associated with cardiovascular risk factors. Our findings speak against categorizing FA based on physicochemical properties. Circulating MUFAs may warrant further investigation as prognostic markers in HFpEF.

## 1. Introduction

Heart failure with preserved ejection fraction (HFpEF) is a heterogeneous clinical syndrome with different aetiologies [1]. Its prevalence rises continuously due to the aging population and increasing rates of cardiometabolic disease [2]. In individuals with metabolic disorders, such as type 2 diabetes mellitus (T2D) and hypertension, obesity-related HFpEF is a common phenotype [3]. In this subgroup of HFpEF patients, the comprehensive treatment of comorbidities or cardiometabolic risk factors has prognostic relevance [3,4].

Monounsaturated fatty acids (MUFAs) are a heterogeneous group of fatty acids. They occur in several foods, such as olive oil, avocados, and nuts [5]. Notably, some MUFAs, in particular, palmitoleic acid (C16:1n7) [6] and, to a lesser extent, oleic acid (C18:1n9), are biomarkers for the endogenous de novo synthesis of fatty acids from dietary starch, sugar, protein, and alcohol. This metabolic pathway is called hepatic de novo lipogenesis (DNL). It becomes relevant in an elevated hepatic triglyceride pool (i.e., fatty liver disease) due to nutrient overabundance or malnutrition [6,7].

Red-blood-cell (RBC) MUFAs or whole blood MUFAs are reliable indicators for cardiac and other tissue MUFA levels. Compared to subjective memory-based methods of assessing food intake, blood MUFAs are an objective biomarker [8]. In that regard, blood MUFAs reflect intake, endogenous production, distribution volume, and catabolism of MUFAs over the last three months [9,10]. This is an essential aspect, since an excess of MUFAs, such as C16:1n7 with potentially adverse health effects, is more related to endogenous overproduction than to dietary MUFA intake per se [7,11,12].

In that regard, circulating blood levels of individual MUFAs in plasma or erythrocytes were differentially linked to incident heart failure (HF) [11], cardiometabolic traits associated with HFpEF such as non-alcoholic-fatty liver disease (NAFLD) or decreased insulin sensitivity [6], and all-cause mortality [13,14] in previous analyses.

Regarding incident HF, Lee et al. reported that changes in C16:1n9 and C18:1n7 were associated with incident HF [11]. Regarding all-cause mortality, Lai et al. reported an association with higher long-term circulating MUFAs C16:1n7 and C18:1n9 [14]. Conversely, Delgado et al. reported an increased risk of all-cause mortality in very high-risk individuals whose levels of oleic acid, gondoic acid, and nervonic acid were increased by one standard deviation (SD) [13].

Overall, contradictory results have been reported regarding the association between distinct MUFAs and health outcomes, including incident HF. Of note, even within the same outcomes, such as all-cause mortality, contradictory evidence exists [13,14]. MUFAs thus are a biologically heterogeneous group of FAs. Furthermore, methodological issues (measurements in different compartments such as erythrocytes, whole blood, plasma, and plasma phospholipids) might explain the disparate associations of MUFAs and health outcomes or surrogates for these outcomes.

Specifically, in patients with HFpEF, the associations between blood MUFA levels and patient characteristics are unknown. We therefore conducted an exploratory analysis of individual MUFA whole blood levels in 404 HFpEF patients from the Aldosterone in Diastolic Heart Failure (Aldo-DHF) trial. This analysis investigated associations between circulating MUFAs with cardiometabolic traits, submaximal and maximal aerobic capacity, left ventricular diastolic function, and neurohumoral activation.

## 2. Methods

### 2.1. Study Design

This is a secondary analysis of the Aldo-DHF trial (ISRCTN 94726526). We analysed MUFA levels in blood retrospectively. Eighteen blood aliquots were unavailable due to storage loss, as described previously [15].

### 2.2. The Aldo-DHF Trial

The Aldo-DHF trial evaluated the effect of aldosterone-receptor blockade on diastolic function (E/e′) and VO2 peak in patients with HFpEF for 12 months. The design was multicentric, prospective, double-blind, and placebo-controlled. Inclusion criteria were age >50 years, New York Heart Association (NYHA) class II or III symptoms, left ventricular ejection fraction (LVEF) ≥50%, maximal exercise capacity (VO2 peak) ≤25 mL/kg/min, and diastolic dysfunction on echocardiography (grade 1) or atrial fibrillation at presentation [16]. Echocardiographic evaluation of diastolic function was conducted according to the American Society of Echocardiography guidelines [16].

#### Laboratory Measurements

- Laboratory methods: Aldo-DHF Trial

Venous blood samples (non-fasting) were taken at baseline and after 12 mFU according to study protocol as described previously [16]. Samples were immediately cooled and stored at −80 °C (−112 °F) [16].

- Laboratory methods: HS-Omega-3 Index^®^ methodology

In order to maintain stable FA levels, samples were immediately cooled, centrifugated, and processed for storage at −80 °C (−112 °F). 2.0 mL aliquots of frozen (−80 °C) EDTA blood taken at baseline were transported to Omegametrix (Martinsried, Germany) and analysed according to the HS-Omega-3 Index^®^ methodology, which recalculates the long-chain omega-3 fatty acid content EPA&DHA into the erythrocyte Omega-3 Index. MUFAs are given as relative amounts of palmitoleic acid (C16:1n7), oleic acid (C18:1n9), eicosenoic acid (C20:1n9), and nervonic acid (C24:1n9) expressed as a percentage of 26 fatty acids in the blood [17]. Acid transesterification generated fatty acid methyl esters. These were analysed by gas chromatography. A GC2010 Gas Chromatograph (Shimadzu, Duisburg, Germany) with an SP2560, 100-m column (Supelco, Bellefonte, PA, USA) using hydrogen as the carrier gas was used. We identified fatty acids by comparing them with a standard mixture of fatty acids. The analyses were quality-controlled according to DIN ISO 15189.

### 2.3. Ethics

The Aldo-DHF Trial was conducted in accordance with the Declaration of Helsinki and the principles of good clinical practice. All responsible ethics committees agreed with the protocol. The study participants gave written informed consent prior to study-related procedures.

### 2.4. Statistical Analysis

Mean ± standard deviation (SD) with a 95% confidence interval or as the median and interquartile range (IQR) were used to present continuous variables. Categorical data are reported in absolute numbers and percentages. For a description of the association of MUFAs with patient characteristics (cardiometabolic phenotype, aerobic capacity, left ventricular diastolic function, and neurohumoral activation at baseline and at 12 mFU), we used Spearman’s correlation coefficient. All correlations were adjusted for markers of truncal adiposity (waist circumference and waist-to-height ratio), body-mass-index, and HbA1c at baseline and 12 mFU. Furthermore, multiple linear regression analyses, using sex and age as covariates, were conducted. All tests were hypothesis-generating without confirmatory interpretation. Therefore, we did not apply a correction for multiple testing. R and RStudio, version R-4.2.125 (R Foundation for Statistical Computing, Vienna, Austria) were used for statistical analyses. Statistical significance was defined by a *p*-value < 0.05.

## 3. Results:

### 3.1. Study Population

**Figure 1:**
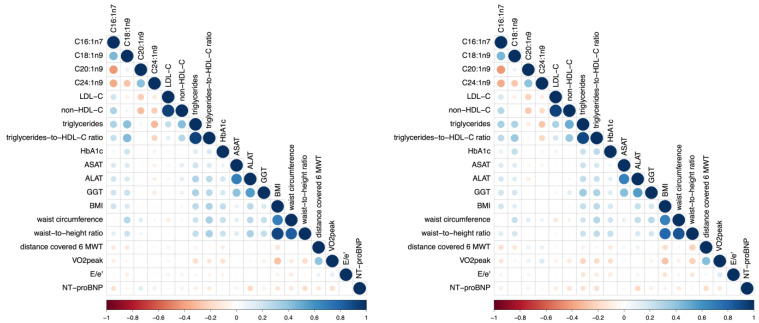
Correlation plot of selected MUFAs and patient characteristics at baseline (**left**) and after 12 mFU (**right**). The blue colour characterizes a positive association; the red colour characterizes an inverse association. Higher colour intensity and larger circles denote a stronger association; lower colour intensity and smaller circles denote a weaker association. Abbreviations: C16:1n7 (palmitoleic acid), C18:1n9 (oleic acid), C20:1n9 (eicosenoic acid), C24:1n9 (nervonic acid), LDL-C (low-density lipoprotein-cholesterol), non-HDL-C (non-high-density lipoprotein-cholesterol), HbA1c (hemoglobin A1c), ASAT (aspartate aminotransaminase), ALAT (alanine aminotransaminase), GGT (γ-glutamyltransferase), BMI (body-mass-index), 6 MWT (6 Minute Walk Test, i.e., sub-maximal exercise test), VO2 peak (maximum exercise capacity), E/e′ (diastolic function), NT-proBNP (N-terminal pro–brain-type natriuretic peptide).

Baseline characteristics are shown in Table 1. Figure 1 illustrates the associations of individual MUFAs with patient characteristics. Table 2 and Table 3 show correlations of individual MUFAs at baseline (C16:1n7, C18:1n9, C20:1n9, C24:1n9) and patient characteristics at baseline (Table 2) and after 12 mFU (Table 3). Significant associations between individual MUFAs and patient characteristics are reported below. Adjusting all correlations for markers of truncal adiposity (waist circumference and waist-to-height ratio), body-mass-index, and HbA1c at baseline and 12 mFU did not alter the overall pattern of the associations described. The adjusted correlations are shown in Supplemental Tables S1 and S2.

Linear regression analyses were additionally conducted to describe the association of MUFAs with patient characteristics at baseline and at 12 mFU as depicted in Supplemental Tables S3 and S4. These models showed the same pattern of associations between MUFAs and patient characteristics; therefore, β-coefficients and *p*-values are not explicitly reported in the text. All models were adjusted using sex and age as a covariate. Both sex and age had a significant influence in several models; however, this did not skew the results in a specific direction regarding fatty acids.

### 3.2. Analysis of Individual MUFAs

- Palmitoleic acid (C16:1n7)

**Figure 2:**
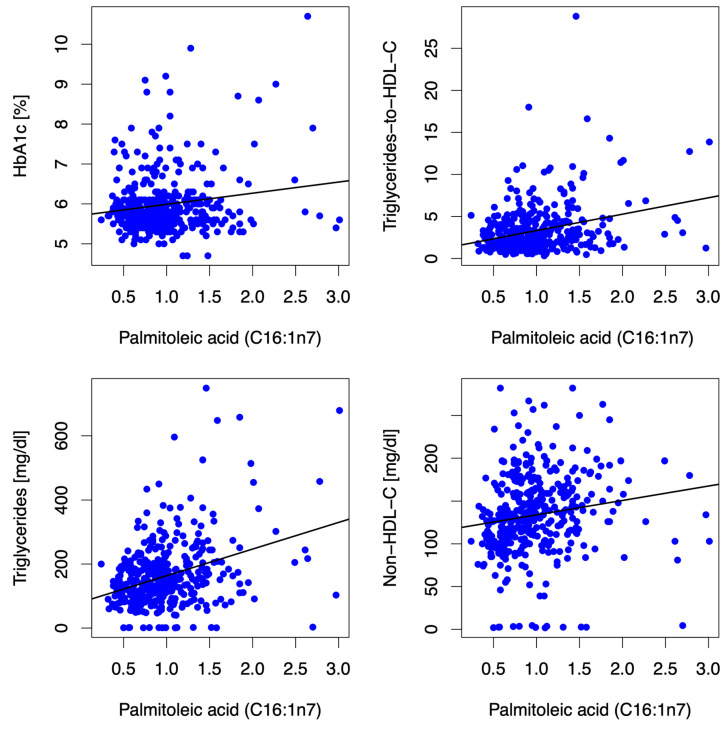
Scatter plots depict correlations between the MUFA C16:1n7 and HbA1c, triglycerides-to-HDL-C ratio, triglycerides, and non-HDL-C at baseline. The MUFA C16:1n7 (expressed as % of a total of 26 identified FAs in the blood) was positively associated with HbA1c (r = 0.05, *p* = 0.333), triglycerides-to-HDL-C-Ratio (r = 0.23, *p* < 0.001), triglycerides (r = 0.317, *p* < 0.001), non-HDL-C (r = 0.311, *p* < 0.001) and LDL-C [(r = 0.201, *p* < 0.001) not depicted in the figure] at baseline. Abbreviations: HbA1c (hemoglobin A1c), HDL-C (high-density lipoprotein-cholesterol), non-HDL-C (non-high-density lipoprotein-cholesterol), LDL-C (low-density lipoprotein-cholesterol).

Higher blood levels of palmitoleic acid correlated with adiposity/truncal adiposity [body-mass-index (r = 0.208, *p* < 0.001), waist-to-height ratio (r = 0.11, *p* = 0.034)] and associated clinical traits such as markers for atherogenic dyslipidemia [triglyceride-to-HDL-C-ratio (r = 0.23, *p* < 0.001), triglycerides (r = 0.317, *p* < 0.001), non-HDL-C (r = 0.311, *p* < 0.001)], LDL-C (r = 0.201, *p* < 0.001), and liver enzymes [aspartate aminotransferase (ASAT) (r = 0.161, *p* = 0.002), alanine aminotransferase (ALAT) (r = 0.196, *p* < 0.001), gamma-glutamyl transferase (GGT) (r = 0.201, *p* < 0.001)] at baseline and after 12 mFU, as depicted in Figure 2. Although weak, these associations were consistent overall and followed a biologically highly plausible pattern. Higher circulating palmitoleic acid was associated with lower submaximal functional capacity (i.e., distance covered in the 6 MWT) (r = −0.126, *p* = 0.015) at baseline and after 12 mFU. No significant associations (i.e., consistent significant association/directionality at baseline and 12 mFU) between palmitoleic acid and either left ventricular diastolic function (E/e′) or neurohumoral activation (NT-proBNP) were observed.

- Oleic acid (C18:1n9)

Blood levels of oleic acid correlated positively with HbA1c (r = 0.209, *p* < 0.001), atherogenic dyslipidemia [triglycerides-to-HDL-C ratio (r = 0.436, *p* < 0.001), triglycerides (r = 0.418, *p* < 0.001)], body-mass-index (r = 0.165, *p* < 0.001), truncal adiposity [waist circumference (r = 0.28, *p* < 0.001), waist-to-height ratio (r = 0.215, *p* < 0.001)], liver enzymes [ALAT (r = 0.166, *p* = 0.001), ASAT (r = 0.159, *p* = 0.002), GGT (r = 0.225, *p* < 0.001)], and correlated inversely with submaximal aerobic capacity (distance covered in 6MWT) (r = −0.122, *p* = 0.019) at baseline/12 mFU. No significant associations (i.e., consistent significant association/directionality at baseline and 12 mFU) between oleic acid and either left ventricular diastolic function (E/e′) or neurohumoral activation (NT-proBNP) were observed.

- Eicosenoic acid (C20:1n9)

Higher levels of circulating eicosenoic acid correlated positively with waist circumference (r = 0.134, *p* = 0.01) and correlated inversely with non-HDL-C (r = −0.304, *p* < 0.001), as well as LDL-C (r = −0.25, *p* < 0.001) at baseline/12 mFU. Furthermore, we observed an inverse association with body-mass-index (r = −0.032, *p* < 0.001) at baseline and an inverse association with triglycerides (r = −0.114, *p* = 0.035) after 12 mFU. No significant associations (i.e., consistent significant association/directionality at baseline and 12 mFU) between eicosenoic acid and either left ventricular diastolic function (E/e′) or neurohumoral activation (NT-proBNP) were observed.

- Nervonic acid (C24:1n9)

**Figure 3:**
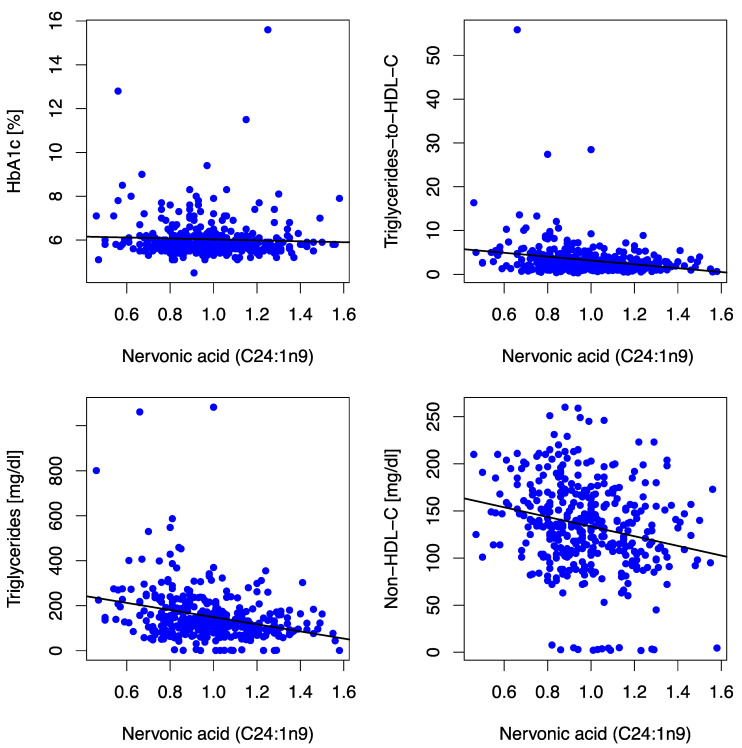
Scatter plots depict correlations between the MUFA C24:1n9 and HbA1c, triglycerides-to-HDL-C ratio, triglycerides, and non-HDL-C at baseline. The MUFA C24:1n9 (expressed as % of a total of 26 identified FAs in blood) was inversely associated with HbA1c (r = −0.072, *p* = 0.169), triglycerides-to-HDL-C-Ratio (r = −0.215, *p* < 0.001), triglycerides (r = −0.323, *p* < 0.001), non-HDL-C (r = −0.244, *p* < 0.001) and LDL-C [(r = −0.123, *p* = 0.018) not depicted in the figure] at baseline. Abbreviations: HbA1c (hemoglobin A1c), HDL-C (high-density lipoprotein-cholesterol), non-HDL-C (non-high-density lipoprotein-cholesterol), LDL-C (low-density lipoprotein-cholesterol).

Nervonic acid correlated inversely with surrogate markers for atherogenic dyslipidemia [triglyceride-to-HDL-C ratio (r = −0.215, *p* < 0.001), triglycerides (r = −0.323, *p* < 0.001), non-HDL-C (r = −0.244, *p* < 0.001)], and LDL-C (r = −0.123, *p* = 0.018) at baseline/12 mFU, as illustrated in Figure 3. Furthermore, we observed an inverse association with body-mass-index (r = −0.032, *p* < 0.001) and GGT (r = −0.116, *p* = 0.026) at baseline. No significant associations (i.e., consistent significant association/directionality at baseline and 12 mFU) between nervonic acid and either left ventricular diastolic function (E/e′) or neurohumoral activation (NT-proBNP) were observed.

## 4. Discussion

### 4.1. Principal Findings

We evaluated the associations of circulating levels of individual MUFA proportions with patient characteristics (cardiometabolic phenotype, functional capacity, left ventricular diastolic function on echocardiography, and neurohumoral activation) in 404 patients with HFpEF from the Aldo-DHF trial.

The principal finding from this exploratory analysis is that individual blood MUFAs are heterogeneous regarding their association with HFpEF patient characteristics. We observed that higher circulating levels of the two MUFAs, palmitoleic acid (C16:1n7) and oleic acid (C18:1n9), which both indicate endogenous fatty acid synthesis, were associated with clinical traits of the obesity-related HFpEF phenotype. Conversely, the MUFAs eicosenoic acid (C20:1n9) and nervonic acid (C24:1n9) were associated with a less pronounced risk profile in HFpEF patients, as depicted in the graphical abstract. These findings add to the literature on the heterogeneity of fatty acids regarding associations with health within distinct physicochemical groups, such as saturated fatty acids [18], trans fatty acids [19], and n-3 polyunsaturated fatty acids [15], in patients with HFpEF. Based on our findings, caution may be warranted when using generalizing terms such as ‘‘monounsaturated fatty acids’’. Instead, consideration of individual MUFAs may be more helpful regarding associations with the cardiometabolic phenotype.

### 4.2. Individual MUFAs and Patient Characteristics

Blood MUFAs are a biomarker for MUFA levels in the myocardium and other body compartments over three months. MUFA blood levels reflect intake, endogenous production, distribution volume, and catabolism. The mean percentages of C16:1n7 (palmitoleic acid), C18:1n9 (oleic acid), C20:1n9 (eiconsenoic acid), and C24:1n9 (nervonic acid) in the Aldo-DHF cohort were 0.97%, 17.83%, 0.23% and 0.98%, respectively (given as % of 26 identified FAs in whole blood). No comparable data exist on whole blood MUFA concentrations in HFpEF patients. In a cohort of patients hospitalized for systolic heart failure (HFrEF), Berliner et al. reported higher levels of palmitoleic acid (1.77%) and oleic acid (23.17%) and lower levels of nervonic acid (0.31%) compared to our patients with clinically stable HFpEF. [20] Whole blood levels of eiconsenoic acid (0.23%) in the HFrEF population were identical to the levels observed in our cohort of HFpEF patients. [20] In that regard, it is worth noting that the Omega-3 Index (EPA + DHA) in patients hospitalized for systolic heart failure was markedly lower (3.7 ± 1.0%) [20] than that in our Aldo-DHF cohort with clinically stable HFpEF (5.7 ± 1.7%) [15]. These observations may overall either speak to different underlying mechanisms in HFrEF and HFpEF or the observation that the HFrEF population studied by Berliner et al. was more late-stage/decompensated HF, which is consistent with the difference in NYHA class in the two studies (43% NYHA class III or IV in the HFrEF population studied by Berliner et al. [20] compared to 87% NYHA class II but only 13% NYHA class III in our cohort of HFpEF patients [15]).

- De novo lipogenesis-related MUFAs palmitoleic acid and oleic acid

We observed a positive association of the MUFA palmitoleic acid at baseline and after 12 mFU with cardiovascular risk factors or clinical traits of obesity-related HFpEF; LDL-C and non-HDL-C [21], triglycerides and the triglyceride-to-HDL-C ratio, the latter being a metabolic marker for high-risk coronary plaque [22,23,24], and anthropometric markers indicative truncal adiposity [25] and adiposity. The liver enzymes ALAT and GGT, [26,27] which may indicate NAFLD and MetS, were positively associated with palmitoleic acid, as was ASAT. The biological plausibility underlying our findings may be conferred by the functional role of palmitoleic acid in metabolism. Palmitoleic acid is formed by elongation of palmitic acid (C16:0), the most abundant SFA in the human body and is largely synthesized endogenously via hepatic DNL [7]. A positive energy balance, a carbohydrate, in particular fructose, intake above requirements [28], a lifestyle low in physical activity and conditions related to these risk factors, such as NAFLD, MetS and T2DM, upregulate DNL [7,29]. This can result in high tissue content of C16:0 and C16:1n7, the latter being an objective lipid biomarker for lipogenesis and elevated hepatic triglyceride pool [6]. Furthermore, circulating (DNL-related) FAs such as C16:0, C16:1n7, and C18:1n9 increase inflammation [30] and modulate critical pathways associated with insulin resistance, metabolic dysregulation [31], and lipogenesis [14], which are involved in the pathophysiology of HFpEF. Overall, these biological properties may underlie the observation that circulating palmitoleic acid has been linked to all-cause mortality [14] and incident HF [11]. Furthermore, in the Framingham Offspring Cohort, in individuals in their mid-60s during follow-up for 11 years, RBC percentage of palmitoleic acid was directly linked with total mortality risk [32]. Regarding incident HF, Lee et al. observed that higher habitual levels of palmitic acid and increasing levels over time of C16:0 were associated with incident HF in individuals from The Cardiovascular Health Study [11]. However, they did not observe an association with incident HF with C16:1n7 [11]. In one of our previous analyses, in line with the work of Lee et al., C16:0 was associated with an unfavorable cardiometabolic phenotype in patients with HFpEF [18]. These studies, however, are not directly comparable due to different study populations. First, while we investigated a cohort of individuals living with HFpEF, Lee et al. had information about HF-phenotype in only 60% of the cohort [HFrEF (EF < 40%, n = 295) and HFpEF (EF > 50%, n = 402)] [11], second, different methodologies were used to measure circulating MUFAs and third, differing study designs were used (incident vs. manifest HF). In our analysis, C16:1n7 was associated with a lower submaximal functional capacity. This is an established risk indicator for adverse outcomes in HFpEF. C16:1n7 was, however, not associated with left ventricular dysfunction on echocardiography or NT-proBNP. A Pub Med search yielded no data on the associations of C16:1n7 and functional capacity. Interestingly, in athletes, levels of palmitoleic acid (C16:1n7) in serum were associated with a higher diastolic interventricular septum thickness and palmitoleic acid has hence been discussed as a molecular comediator of pathological exercise-induced cardiac hypertrophy or HF by the authors [33]. An alternative hypothesis based on more recent evidence is that C16:1n7 may be a marker of a high-risk metabolic phenotype and associated cardiometabolic traits such as arterial hypertension or left ventricular hypertrophy. Overall, in line with its functional role as a marker of de novo lipogenesis if overnutrition is present [6], and prior work regarding metabolic dysfunction and all-cause mortality [14,31], C16:1n7 was associated with a higher-risk cardiometabolic phenotype in our cohort of HFpEF patients.

Similar to palmitoleic acid, we observed a positive correlation between oleic acid and established cardiovascular risk factors such as dysglycemia, hypertriglyceridaemia/atherogenic dyslipidemia, and truncal adiposity in the Aldo-DHF cohort. Furthermore, liver enzymes suggestive of NAFLD (i.e., ALAT [27] and GGT [26]) correlated with C18:1n9. Oleic acid is found in healthy foods such as avocados, seeds, nuts, and olive oil. However, these healthy foods might confer benefits due to other constituents (e.g., polyphenols and fibre) rather than their content in MUFAs per se [34,35]. Like palmitoleic acid, oleic acid is endogenously synthesized in the hepatic de novo lipogenesis pathway. Evidence is heterogeneous concerning its association with HF, cardiometabolic risk factors, and cardiovascular/all-cause mortality. While some studies have shown lower all-cause mortality, cardiovascular mortality, cardiovascular events, and stroke, other studies have shown an elevated risk for acute myocardial infarction with higher levels of oleic acid; for example, erythrocyte levels of oleic acid showed a direct association with mortality [13] and correlated directly with inflammatory burden, endothelial activation, and HF in patients from The Ludwigshafen Risk and Cardiovascular Health (LURIC) Study [13]. Aligning with these findings, over 6500 patients from the Multi-Ethnic Study of Atherosclerosis (MESA) showed significantly greater risks of incident HF, cardiovascular disease (CVD) and all-cause mortality with higher circulating oleic acid levels in plasma [36]. Others, such as Morin et al., who analysed the relationship between plasma phospholipid oleic acid levels and HF risk in individuals (788 incident HF cases; 788 controls) from the Physicians’ Health Study, did not find a significant association between circulating oleic acid levels and HF in men [37]. Similarly, Lee et al. observed no association between plasma phospholipid C18:1n9 levels and incident HF in 4249 participants from The Cardiovascular Health Study [11]. Overall, there is significant heterogeneity, possibly due to the different methodologies used to measure fatty acids, i.e., erythrocyte levels in the LURIC cohort [13] versus plasma phospholipid oleic acid levels in the studies with neutral results (Physicians’ Health Study [37] and The Cardiovascular Health Study [11]) or different patient populations (patients with very high cardiovascular risk [13] versus solely male participants in the Physicians’ Health Study [37] versus older individuals in The Cardiovascular Health Study [11]). Regarding methodological issues, it is worth noting that plasma phospholipid fatty acid levels, compared to erythrocyte levels or whole blood levels, have higher biological and analytical variability, worse signal-to-noise-ratio, and, overall, less-robust data [10,38]. There is neither evidence regarding associations of circulating oleic acid levels and incident HFpEF nor is there evidence regarding associations of circulating oleic acid levels and prognosis in patients with pre-existing HFpEF. Our data are, therefore, novel.

- The MUFAs eicosenoic acid and nervonic acid

We observed that higher levels of circulating eicosenoic acid and nervonic acid correlated inversely with surrogate markers for atherogenic dyslipidemia and body-mass-index. There is no literature to date regarding the associations of eicosenoic acid with incident HF or cardiometabolic risk factors. The terms “eicosenoic acid and heart failure”, “C20:1n9 and heart failure” and “eicosenoic acid and cardiometabolic risk factors” did not yield any pub med result (pub med search as of 25 April 2023). One piece of literature describes associations of nervonic acid with cardiometabolic risk, HF, and mortality. Delgado et al. observed that in very high-risk patients from The LURIC Study, nervonic acid in erythrocytes correlated inversely with LDL-C, aligning with our findings, but showed a direct association with mortality and with inflammatory burden, endothelial activation and HF [13]. The known physiological function of nervonic acid is its role in synthesizing nerve cell myelin, providing a rationale for its use as a dietary supplement treating neurological conditions involving demyelination such as multiple sclerosis [39]. However, its functional role in metabolism or, more specifically, cardiometabolism, is unknown, and except the LURIC study, the terms “nervonic acid and heart failure” and “C24:1n9 and heart failure” did not yield any pub med result (pub med search as of 25 April 2023). This again underscores the novelty of our work and—given the observed direct association with HF in LURIC—further underpins the necessity to investigate fatty acid-based biomarkers in patients with HF or HFpEF specifically.

- MUFAs and left ventricular diastolic function/neurohumoral activation

No consistent patterns were found between left ventricular dysfunction and NT-proBNP and blood MUFAs. This aligns with our previous analyses on the associations of individual saturated FA, trans FA, and n-3 FA and echocardiographic markers of left ventricular diastolic function [15,18,19].

## 5. Strengths and Limitations

This analysis is not without limitations. First, the Aldo-DHF cohort was Caucasian. Therefore, our findings may not apply to non-Caucasian individuals. Second, fatty acids were measured only in baseline samples. In that regard, it is worth noting that MUFAs in the blood are not constant but may change (i.e., due to changes in diet or medical conditions). Third, no specific information on the relative determinants driving blood MUFA concentrations in this population is available. Finally, no data are available on hard clinical endpoints.

A significant strength is the comprehensive phenotypic characterization of the Aldo-DHF cohort, which comprised 404 patients living with HFpEF. Moreover, 53% of the patients included in Aldo-DHF were female. This is representative of the gender distribution that would be expected in a cohort of patients living with HFpEF. [1] Finally, our findings are novel since no analysis of whole blood MUFAs in patients with HFpEF has been done. It is also worth noting that whole blood FAs have advantages over (a) the assessment of FA intake via subjective memory-based methods (i.e., food frequency questionnaires); and (b) over measuring MUFA levels in other lipid compartments such as plasma. Regarding the latter, whole blood levels measured with the HS-Omega-3 Index methodology have lower biological and analytical variability than those measured e.g., in plasma [10]. Regarding the further, the assessment of associations between FA and risk markers using subjective memory-based methods as opposed to measuring objective biomarkers in blood has been criticized due to the possibility of implausible data [8].

## 6. Translational Outlook

Prognosis in HFpEF, particularly in obesity-related HFpEF, is determined by the treatment of comorbidities and optimal risk-factor control [4]. We found that individual blood MUFAs have distinct associations with higher-/lower expression of obesity-related HFpEF traits. Aligning with previous work, which points to a potential role of DNL-related MUFAs as biomarkers for the presence of hepatic steatosis or metabolic dysfunction [6,40], all-cause mortality [14] and cardiac hypertrophy [33], we showed that circulating levels of palmitoleic and oleic acid, which are both markers for DNL [7,12], were associated with obesity-related HFpEF characteristics. In contrast, the two circulating MUFAs, eicosenoic and nervonic acid, were associated with a lower expression of HFpEF-relevant risk factors. In previous analyses regarding the association of specific fatty acids in the DNL pathway and incident HF/all-cause mortality, higher habitual levels and increases in DNL-associated fatty acids were independently linked to an increased risk of HF [11,14]. This is the first analysis of circulating (DNL-associated) MUFAs in patients with HFpEF. The findings from this exploratory analysis warrant further investigation to explore potentially relevant new risk pathways in HFpEF. As discussed previously by Lee et al., downregulation of the de novo lipogenesis pathway by minimizing dietary refined starch, sugars, and alcohol could be targeted to reduce the risk of HF or ameliorate prognosis if these associations prove causal [11].

## 7. Conclusions

In HFpEF patients, higher blood levels of the MUFAs palmitoleic and oleic acid were associated with cardiometabolic risk factors. In contrast, the MUFAs eicosenoic and nervonic acid were associated with a lower expression of HFpEF-relevant risk factors. Overall, our results speak against using generalizing terms such as monounsaturated fatty acids and indicate that caution should be used when using the physicochemical properties of fatty acids to categorize them regarding their health effects. Furthermore, an investigation of fatty acid-based prognostic biomarkers may be warranted in HFpEF.

## Figures and Tables

**Table 1:** Baseline Characteristics. Abbreviations: SD (standard deviation), HbA1c (hemoglobin A1c), LDL-C (low-density lipoprotein-cholesterol), HDL-C (high-density lipoprotein-cholesterol), non-HDL-C (non-high density lipoprotein-cholesterol), NT-proBNP (N-terminal pro-brain-type natriuretic peptide), IQR (interquartile range), MUFAs (monounsaturated fatty acids), C16:1n7 (palmitoleic acid), C18:1n9 (oleic acid), C20:1n9 (eicosenoic acid), C24:1n9 (nervonic acid), NYHA (New York Heart Association), ACE (angiotensin-converting enzyme), LV (left ventricular), A (peak atrial transmitral ventricular filling velocity), e′ (early diastolic tissue Doppler velocity), E (peak early transmitral ventricular filling velocity). ^a^ Data are expressed as no. (%) unless otherwise specified. ^b^ 2 missing from analysis (n  =  402). ^c^ Body-mass-index is defined as weight in kilograms divided by height in meters squared.

Baseline Characteristics ^a^	Total (n = 404)
Demographics	
Age, mean (SD), y	67 (8)
Female	212 (53)
Laboratory measures	
HbA1c (%)	6.0 (0.8)
LDL-C (mg/dL)	117 (42)
HDL-C (mg/dL)	56 (18)
Triglycerides (mg/dL)	161 (103)
non-HDL-C (mg/dL)	133 (47)
Triglycerides-to-HDL-C ratio	3.3 (2.8)
NT-proBNP, median (IQR), ng/L	158 (82–298)
MUFAs (%) C16:1n7 C18:1n9 C20:1n9 C24:1n9	0.97 (0.43) 17.83 (2.20) 0.23 (0.04) 0.98 (0.213)
Medical history	
Hospitalization for heart failure in past 12 months ^b^	149 (37)
Hypertension	370 (92)
Diabetes mellitus	66 (16)
Atrial fibrillation	65 (16)
Physical examination, mean (SD)	
Body-mass-index ^c^	28.9 (3.6)
Waist Circumference, (cm) In Men In Women	98.1 (11.0) 103.7 (9.0) 93.1 (10.3)
Waist-to-height ratio	0.49 (0.1)
Systolic blood pressure, mm Hg	135 (18)
Diastolic blood pressure, mm Hg	79 (11)
Heart rate,/min	66 (11)
Signs and symptoms	
NYHA functional class	
II	350 (87)
III	54 (13)
Peripheral edema	160 (40)
Nocturia	325 (80)
Paroxysmal nocturnal dyspnea	66 (16)
Nocturnal cough	61 (15)
Fatigue	241 (60)
Current medications	
ACE inhibitors/angiotensin receptor antagonists	310 (77)
Beta-blockers	290 (72)
Diuretics	213 (53)
Calcium antagonists	97 (24)
Lipid-lowering drugs	221 (55)
Echocardiography, mean (SD)	
LV ejection fraction, %	68 (8)
LV diameter (end-diastolic), mm	46.5 (6.2)
LV diameter (end-systolic), mm	25.3 (6.4)
LV mass index, g/m^2^	114.15 (45.53)
Left atrial volume index, mL/m^2^	43.1 (41.6)
E-wave velocity, cm/s	73 (20)
Medial e′ wave velocity, cm/s	5.9 (1.3)
E/e′	7.1 (1.5)
E/A velocity ratio	0.91 (0.33)
Isovolumic relaxation time, ms	88 (26)
Deceleration time, ms	243 (63)
Grade of diastolic dysfunction, No. (%)	
I	295 (73)
II	81 (20)
III	4 (1)
IV	3 (1)

**Table 2:** Correlations between circulating MUFAs and patient characteristics at baseline. Abbreviations: LDL-C (low-density lipoprotein-cholesterol), non-HDL-C (non-high-density lipoprotein-cholesterol), HbA1c (hemoglobin A1c), ASAT (aspartate aminotransaminase), ALAT (alanine aminotransaminase), GGT (γ-glutamyltransferase), BMI (body-mass-index), 6 MWT (6 Minute Walk Test, i.e., sub-maximal exercise test), VO2 peak (maximum exercise capacity), E/e′ (diastolic function), NT-proBNP (N-terminal pro–brain-type natriuretic peptide). Significant values are in bold. ^§^ All tests were performed two-sided. r * (Spearman’s correlation coefficient).

		C16:1n7	C18:1n9	C20:1n9	C24:1n9
LDL-C	r * p ^§^	**0.201** **<0.001**	−0.088 0.089	**−0.25** **<0.001**	**−0.123** **0.018**
non-HDL-C	r * p ^§^	**0.311** **<0.001**	0.055 0.292	**−0.304** **<0.001**	**−0.244** **<0.001**
triglycerides	r * p ^§^	**0.317** **<0.001**	**0.418** **<0.001**	−0.059 0.261	**−0.323** **<0.001**
triglycerides-to-HDL-C ratio	r * p ^§^	**0.23** **<0.001**	**0.436** **<0.001**	0.016 0.765	**−0.215** **<0.001**
HbA1c	r * p ^§^	0.05 0.333	**0.209** **<0.001**	0.016 0.759	−0.072 0.169
ASAT	r * p ^§^	**0.161** **0.002**	**0.159** **0.002**	−0.006 0.903	−0.088 0.091
ALAT	r * p ^§^	**0.196** **<0.001**	**0.166** **0.001**	−0.014 0.79	−0.094 0.071
GGT	r * p ^§^	**0.201** **<0.001**	**0.225** **<0.001**	0.024 0.641	**−0.116** **0.026**
BMI	r * p ^§^	**0.208** **<0.001**	**0.165** **0.001**	−0.032 0.541	−0.032 0.541
waist circumference	r * p ^§^	0.01 0.85	**0.28** **<0.001**	0.134 0.01	0.056 0.281
waist-to-height ratio	r * p ^§^	**0.11** **0.034**	**0.215** **<0.001**	0.067 0.196	0.021 0.682
distance covered 6 MWT	r * p ^§^	**−0.126** **0.015**	**−0.122** **0.019**	0.038 0.462	0.034 0.51
VO2 peak	r * p ^§^	−0.101 0.052	−0.052 0.32	−0.017 0.741	0.005 0.92
E/e′	r * p ^§^	−0.03 0.558	**−0.102** **0.049**	−0.016 0.765	0.035 0.496
NT-proBNP	r * p ^§^	**−0.159** **0.002**	−0.053 0.312	**0.131** **0.011**	0.088 0.091

**Table 3:** Correlations of circulating MUFAs and patient characteristics after 12 mFU. Abbreviations: LDL-C (low-density lipoprotein-cholesterol), non-HDL-C (non-high-density lipoprotein-cholesterol), HbA1c (hemoglobin A1c), ASAT (aspartate aminotransaminase), ALAT (alanine aminotransaminase), GGT (γ-glutamyltransferase), BMI (body-mass-index), 6 MWT (6 Minute Walk Test, i.e., sub-maximal exercise test), VO2 peak (maximum exercise capacity), E/e′ (diastolic function), NT-proBNP (N-terminal pro–brain-type natriuretic peptide). Significant values are in bold. ^§^ All tests were performed two-sided. r * (Spearman’s correlation coefficient).

		C16:1n7	C18:1n9	C20:1n9	C24:1n9
LDL-C	r * p ^§^	**0.176** **0.001**	−0.1 0.065	**−0.228** **<0.001**	**−0.108** **0.046**
non-HDL-C	r * p ^§^	**0.287** **<0.001**	0.013 0.814	**−0.268** **<0.001**	**−0.193** **<0.001**
triglycerides	r * p ^§^	**0.321** **<0.001**	**0.308** **<0.001**	**−0.114** **0.035**	**−0.277** **<0.001**
triglycerides-to-HDL-C ratio	r * p ^§^	**0.268** **<0.001**	**0.368** **<0.001**	−0.044 0.421	**−0.21** **<0.001**
HbA1c	r * p ^§^	0.094 0.083	**0.242** **<0.001**	−0.022 0.689	−0.063 0.249
ASAT	r * p ^§^	**0.158** **0.004**	**0.135** **0.012**	−0.055 0.31	−0.063 0.243
ALAT	r * p ^§^	**0.18** **0.001**	**0.136** **0.012**	−0.1 0.065	−0.017 0.761
GGT	r * p ^§^	**0.195** **<0.001**	**0.182** **0.001**	−0.025 0.641	−0.035 0.524
BMI	r * p ^§^	**0.197** **<0.001**	**0.133** **0.014**	−0.001 0.983	0.004 0.941
waist circumference	r * p ^§^	0.024 0.656	**0.239** **<0.001**	**0.133** **0.014**	0.086 0.115
waist-to-height ratio	r * p ^§^	**0.144** **0.008**	**0.181** **<0.001**	0.012 0.826	0.013 0.805
distance covered 6 MWT	r * p ^§^	**−0.17** **0.002**	**−0.137** **0.011**	0.095 0.078	0.101 0.062
VO2 peak	r * p ^§^	**−0.136** **0.012**	−0.046 0.393	0.094 0.084	**0.158** **0.004**
E/e′	r * p ^§^	−0.069 0.206	−0.016 0.766	−0.061 0.264	0.025 0.64
NT-proBNP	r * p ^§^	−0.091 0.206	−0.035 0.516	0.074 0.172	0.044 0.422

## Data Availability

Due to the GDPR regulations and local data protection laws, patient data cannot be provided.

## Supplementary Materials

The following supporting information can be downloaded at: https://www.mdpi.com/article/10.3390/jcm12154938/s1.

## References

[1] Pieske B., Tschöpe C., de Boer R.A., Fraser A.G., Anker S.D., Donal E., Edelmann F., Fu M., Guazzi M., Lam C.S.P. (2019). How to diagnose heart failure with preserved ejection fraction: The HFA–PEFF diagnostic algorithm: A consensus recommendation from the Heart Failure Association (HFA) of the European Society of Cardiology (ESC). Eur. Heart J..

[2] Shah S.J., Kitzman D.W., Borlaug B.A., van Heerebeek L., Zile M.R., Kass D.A., Paulus W.J. (2016). Phenotype-Specific Treatment of Heart Failure with Preserved Ejection Fraction. Circulation.

[3] Seferović P.M., Petrie M.C., Filippatos G.S., Anker S.D., Rosano G., Bauersachs J., Paulus W.J., Komajda M., Cosentino F., de Boer R.A. (2018). Type 2 diabetes mellitus and heart failure: A position statement from the Heart Failure Association of the European Society of Cardiology. Eur. J. Heart Fail..

[4] Shah S.J., Gheorghiade M. (2008). Heart failure with preserved ejection fraction: Treat now by treating comorbidities. JAMA.

[5] Schwingshackl L., Hoffmann G. (2014). Monounsaturated fatty acids, olive oil and health status: A systematic review and meta-analysis of cohort studies. Lipids Health Dis..

[6] Lee J.J., Lambert J.E., Hovhannisyan Y., Ramos-Roman M.A., Trombold J.R., Wagner D.A., Parks E.J. (2015). Palmitoleic acid is elevated in fatty liver disease and reflects hepatic lipogenesis. Am. J. Clin. Nutr..

[7] Carta G., Murru E., Banni S., Manca C. (2017). Palmitic Acid: Physiological Role, Metabolism and Nutritional Implications. Front. Physiol..

[8] Archer E., Hand G.A., Blair S.N. (2013). Validity of U.S. nutritional surveillance:National Health and Nutrition Examination Survey caloric energy intake data, 1971–2010. PLoS ONE.

[9] Harris W.S., Sands S.A., Windsor S.L., Ali H.A., Stevens T.L., Magalski A., Porter C.B., Borkon A.M. (2004). Omega-3 fatty acids in cardiac biopsies from heart transplantation patients: Correlation with erythrocytes and response to supplementation. Circulation.

[10] Harris W.S., Thomas R.M. (2010). Biological variability of blood omega-3 biomarkers. Clin. Biochem..

[11] Lee Y., Lai H.T.M., de Oliveira Otto M.C., Lemaitre R.N., McKnight B., King I.B., Song X., Huggins G.S., Vest A.R., Siscovick D.S. (2020). Serial Biomarkers of De Novo Lipogenesis Fatty Acids and Incident Heart Failure in Older Adults: The Cardiovascular Health Study. J. Am. Heart Assoc..

[12] Qureshi W., Santaren I.D., Hanley A.J., Watkins S.M., Lorenzo C., Wagenknecht L.E. (2019). Risk of diabetes associated with fatty acids in the de novo lipogenesis pathway is independent of insulin sensitivity and response: The Insulin Resistance Atherosclerosis Study (IRAS). BMJ Open Diabetes Res. Care.

[13] Delgado G.E., Krämer B.K., Lorkowski S., März W., von Schacky C., Kleber M.E. (2017). Individual omega-9 monounsaturated fatty acids and mortality-The Ludwigshafen Risk and Cardiovascular Health Study. J. Clin. Lipidol..

[14] Lai H.T.M., de Oliveira Otto M.C., Lee Y., Wu J.H.Y., Song X., King I.B., Psaty B.M., Lemaitre R.N., McKnight B., Siscovick D.S. (2019). Serial Plasma Phospholipid Fatty Acids in the De Novo Lipogenesis Pathway and Total Mortality, Cause-Specific Mortality, and Cardiovascular Diseases in the Cardiovascular Health Study. J. Am. Heart Assoc..

[15] Lechner K., Scherr J., Lorenz E., Lechner B., Haller B., Krannich A., Halle M., Wachter R., Duvinage A., Edelmann F. (2022). Omega-3 fatty acid blood levels are inversely associated with cardiometabolic risk factors in HFpEF patients: The Aldo-DHF randomized controlled trial. Clin. Res. Cardiol..

[16] Edelmann F., Wachter R., Schmidt A.G., Kraigher-Krainer E., Colantonio C., Kamke W., Duvinage A., Stahrenberg R., Durstewitz K., Löffler M. (2013). Effect of spironolactone on diastolic function and exercise capacity in patients with heart failure with preserved ejection fraction: The Aldo-DHF randomized controlled trial. JAMA.

[17] Harris W.S. (2010). The omega-3 index: Clinical utility for therapeutic intervention. Curr. Cardiol. Rep..

[18] Lechner K., von Schacky C., Scherr J., Lorenz E., Bock M., Lechner B., Haller B., Krannich A., Halle M., Wachter R. (2022). Saturated Fatty Acid Blood Levels and Cardiometabolic Phenotype in Patients with HFpEF: A Secondary Analysis of the Aldo-DHF Trial. Biomedicines.

[19] Lechner K., Bock M., von Schacky C., Scherr J., Lorenz E., Lechner B., Haller B., Krannich A., Halle M., Wachter R. (2023). Trans-fatty acid blood levels of industrial but not natural origin are associated with cardiovascular risk factors in patients with HFpEF: A secondary analysis of the Aldo-DHF trial. Clin. Res. Cardiol..

[20] Berliner D., Mattern S., Wellige M., Malsch C., Güder G., Brenner S., Morbach C., Deubner N., Breunig M., Kiefl R. (2019). The omega-3 index in patients with heart failure: A prospective cohort study. Prostaglandins Leukot Essent Fat. Acids.

[21] Mach F., Baigent C., Catapano A.L., Koskinas K.C., Casula M., Badimon L., Chapman M.J., De Backer G.G., Delgado V., Ference B.A. (2019). 2019 ESC/EAS Guidelines for the management of dyslipidaemias: Lipid modification to reduce cardiovascular risk: The Task Force for the management of dyslipidaemias of the European Society of Cardiology (ESC) and European Atherosclerosis Society (EAS). Eur. Heart J..

[22] Lechner K., Halle M. (2019). Are Atherogenic Lipoprotein Phenotype and Inflammation Indicative of Plaque Phenotype and Clinical Stability in Coronary Artery Disease?. JAMA Cardiol..

[23] Vergallo R., Porto I., Crea F. (2019). Are Atherogenic Lipoprotein Phenotype and Inflammation Indicative of Plaque Phenotype and Clinical Stability in Coronary Artery Disease?-Reply. JAMA Cardiol..

[24] Vergallo R., Porto I., D’Amario D., Annibali G., Galli M., Benenati S., Bendandi F., Migliaro S., Fracassi F., Aurigemma C. (2019). Coronary Atherosclerotic Phenotype and Plaque Healing in Patients With Recurrent Acute Coronary Syndromes Compared with Patients with Long-term Clinical Stability: An In Vivo Optical Coherence Tomography Study. JAMA Cardiol..

[25] Ross R., Neeland I.J., Yamashita S., Shai I., Seidell J., Magni P., Santos R.D., Arsenault B., Cuevas A., Hu F.B. (2020). Waist circumference as a vital sign in clinical practice: A Consensus Statement from the IAS and ICCR Working Group on Visceral Obesity. Nat. Rev. Endocrinol..

[26] Banderas D.Z., Escobedo J., Gonzalez E., Liceaga M.G., Ramírez J.C., Castro M.G. (2012). γ-Glutamyl transferase: A marker of nonalcoholic fatty liver disease in patients with the metabolic syndrome. Eur. J. Gastroenterol. Hepatol..

[27] Simental-Mendía L.E., Rodríguez-Hernández H., Rodríguez-Morán M., Guerrero-Romero F. (2012). The alanine aminotransferase to triglycerides ratio as a marker to identify nonalcoholic fatty liver disease. Eur. J. Gastroenterol. Hepatol..

[28] Zhao S., Jang C., Liu J., Uehara K., Gilbert M., Izzo L., Zeng X., Trefely S., Fernandez S., Carrer A. (2020). Dietary fructose feeds hepatic lipogenesis via microbiota-derived acetate. Nature.

[29] Stahl E.P., Dhindsa D.S., Lee S.K., Sandesara P.B., Chalasani N.P., Sperling L.S. (2019). Nonalcoholic Fatty Liver Disease and the Heart: JACC State-of-the-Art Review. J. Am. Coll. Cardiol..

[30] Wu D., Liu J., Pang X., Wang S., Zhao J., Zhang X., Feng L. (2014). Palmitic acid exerts pro-inflammatory effects on vascular smooth muscle cells by inducing the expression of C-reactive protein, inducible nitric oxide synthase and tumor necrosis factor-α. Int. J. Mol. Med..

[31] Mozaffarian D., Cao H., King I.B., Lemaitre R.N., Song X., Siscovick D.S., Hotamisligil G.S. (2010). Circulating palmitoleic acid and risk of metabolic abnormalities and new-onset diabetes. Am. J. Clin. Nutr..

[32] McBurney M.I., Tintle N.L., Vasan R.S., Sala-Vila A., Harris W.S. (2021). Using an erythrocyte fatty acid fingerprint to predict risk of all-cause mortality: The Framingham Offspring Cohort. Am. J. Clin. Nutr..

[33] Foryst-Ludwig A., Kreissl M.C., Benz V., Brix S., Smeir E., Ban Z., Januszewicz E., Salatzki J., Grune J., Schwanstecher A.K. (2015). Adipose Tissue Lipolysis Promotes Exercise-induced Cardiac Hypertrophy Involving the Lipokine C16:1n7-Palmitoleate. J. Biol. Chem..

[34] Astrup A., Magkos F., Bier D.M., Brenna J.T., de Oliveira Otto M.C., Hill J.O., King J.C., Mente A., Ordovas J.M., Volek J.S. (2020). Saturated Fats and Health: A Reassessment and Proposal for Food-Based Recommendations: JACC State-of-the-Art Review. J. Am. Coll. Cardiol..

[35] Mozaffarian D. (2016). Dietary and Policy Priorities for Cardiovascular Disease, Diabetes, and Obesity: A Comprehensive Review. Circulation.

[36] Steffen B.T., Duprez D., Szklo M., Guan W., Tsai M.Y. (2018). Circulating oleic acid levels are related to greater risks of cardiovascular events and all-cause mortality: The Multi-Ethnic Study of Atherosclerosis. J. Clin. Lipidol..

[37] Morin S.J., Gaziano J.M., Djoussé L. (2018). Relation between plasma phospholipid oleic acid and risk of heart failure. Eur. J. Nutr..

[38] Schuchardt J.P., Cerrato M., Ceseri M., DeFina L.F., Delgado G.E., Gellert S., Hahn A., Howard B.V., Kadota A., Kleber M.E. (2022). Red blood cell fatty acid patterns from 7 countries: Focus on the Omega-3 index. Prostaglandins Leukot Essent Fat. Acids.

[39] Sargent J.R., Coupland K., Wilson R. (1994). Nervonic acid and demyelinating disease. Med. Hypotheses.

[40] Volk B.M., Kunces L.J., Freidenreich D.J., Kupchak B.R., Saenz C., Artistizabal J.C., Fernandez M.L., Bruno R.S., Maresh C.M., Kraemer W.J. (2014). Effects of step-wise increases in dietary carbohydrate on circulating saturated Fatty acids and palmitoleic Acid in adults with metabolic syndrome. PLoS ONE.

