# Supplementary material for: De Novo Lipogenesis-Related Monounsaturated Fatty Acids in the Blood Are Associated with Cardiovascular Risk Factors in HFpEF Patients
Source: J Clin Med. 2023 Jul 27;12(15):4938. doi: 10.3390/jcm12154938 (PMC10419368; doi:10.3390/jcm12154938)
Supplement: Supplementary file 1 [file jcm-2525047-supplementary.pdf › jcm-2525047-supplementary.pdf]

Dr. med. Katharina Lechner
Department of Cardiology, German Heart Centre Munich, Technical University Munich
Lazarettstraße 36 · 80636 Munich, Germany
Phone: +49 089 1218 0 · Fax: +49 089 1218 0 · E-mail: katharina.lechner@tum.de

July, 18th 2023

Dr. Emmanuel Andrès, Prof. Dr. Michael G. Hennerici
Editors in Chief, Journal of Clinical Medicine

Dear Dr. Andrès, dear Prof. Hennerici,

We appreciate the opportunity to revise our manuscript entitled "De novo lipogenesis-related monounsaturated fatty acids in the blood are associated with cardiovascular risk factors in HFpEF patients" for consideration as an original article in *Journal of Clinical Medicine*. We would like to thank the reviewers for their insightful comments and efforts towards improving our manuscript. We believe that the manuscript has been strengthened by the revision and hope that our efforts to attend to the reviewer's comments will facilitate the decision to publish our manuscript in *Journal of Clinical Medicine*.

Please find appended our point-by-point response to the reviewers' comments. We have accepted all changes of the previous version (as uploaded in the editorial manager by MDPI) and we have highlighted all new changes.

All authors listed have contributed sufficiently to the manuscript to be included as authors, and all those who are qualified to be authors are listed in the author byline. MH wishes to remove his name (→ see separate Authorship Change Form). MB, JS, EL, BL, AK, RW, AD, FE and KL declare that they have no conflict of interest to disclose with respect to this manuscript. CvS operates Omegametrix, a laboratory for fatty acid analyses. We have included acknowledgements and conflicts of interest after the discussion.

We appreciate your time and are looking forward to your comments.

Kind regards
Katharina Lechner, Matthias Bock and Frank Edelmann, on behalf of the authors

Point-by-point response:

Reviewer #1:

The alterations made contributed to improve the manuscript. Some concerns remain:

We would like to thank the reviewer for his/her thoughtful comments on our manuscript and for his/her efforts towards improving our manuscript. We have extended our analysis and discussion by the issues raised. Below, we offer our point-by-point response to the reviewer's comments.

All data mentioned, including that from multiple linear regression analyses (with and without adjustments for confounding factors), should be presented or, at least, made available as supplementary material.

Thank you for raising this issue. We have provided the data requested (Supplemental Tables 1-4 below) for presentation as (online) supplements if the editors wish. Furthermore, we have summarized these findings in the manuscript.

Supplemental Table S1. Correlations between circulating MUFAs and patient characteristics **at baseline adjusted for body-mass-index, waist circumference, waist-to-height ratio and HbA1c**. Abbreviations: LDL-C (low-density lipoprotein-cholesterol), non-HDL-C (non-high-density lipoprotein-cholesterol), HbA1c (hemoglobin A1c), ASAT (aspartate aminotransaminase), ALAT (alanine aminotransaminase), GGT (γ-glutamyltransferase), BMI (body-mass-index), 6 MWT (6 Minute Walk Test, i.e., sub-maximal exercise test), VO2peak (maximum exercise capacity), E/e´ (diastolic function), NT-proBNP (N-terminal pro–brain-type natriuretic peptide). Significant values are in bold. §All tests were performed 2-sided. r* (Spearman's correlation coefficient).

		C16:1n7	C18:1n9	C20:1n9	C24:1n9
LDL-C	r*	**0.171**	-0.076	**-0.228**	**-0.12**
	p§	**0.001**	0.144	**<0.001**	**0.021**
non-HDL-C	r*	**0.267**	0.058	**-0.283**	**-0.236**
	p§	**<0.001**	0.264	**<0.001**	**<0.001**
triglycerides	r*	**0.291**	**0.393**	-0.061	**-0.323**
	p§	**<0.001**	**<0.001**	0.244	**<0.001**
triglycerides-to-HDL-C ratio	r*	**0.239**	**0.376**	-0.015	**-0.243**
	p§	**<0.001**	**<0.001**	0.767	**<0.001**
ASAT	r*	**0.156**	0.102	-0.021	-0.089
	p§	**0.003**	0.051	0.68	0.086
ALAT	r*	**0.194**	0.072	-0.052	**-0.104**
	p§	**<0.001**	0.169	0.317	**0.046**
GGT	r*	**0.244**	**0.129**	-0.048	**-0.128**
	p§	**<0.001**	**0.013**	0.354	**0.014**
distance covered 6 MWT	r*	-0.059	**-0.147**	-0.016	-0.006
	p§	0.257	**0.004**	0.765	0.902
VO2peak	r*	0.008	-0.036	-0.082	-0.045
	p§	0.886	0.485	0.115	0.392
E/e'	r*	-0.024	-0.076	0.001	0.04
	p§	0.651	0.145	0.986	0.444
NT-proBNP	r*	**-0.154**	0.003	**0.147**	0.096
	p§	**0.003**	0.959	**0.005**	0.064

Supplemental Table S2. Correlations between circulating MUFAs and patient characteristics **after 12 mFU adjusted for body-mass-index, waist circumference, waist-to-height ratio and HbA1c**. Abbreviations: LDL-C (low-density lipoprotein-cholesterol), non-HDL-C (non-high-density lipoprotein-cholesterol), HbA1c (hemoglobin A1c), ASAT (aspartate aminotransaminase), ALAT (alanine aminotransaminase), GGT (γ-glutamyltransferase), BMI (body-mass-index), 6 MWT (6 Minute Walk Test, i.e., sub-maximal exercise test), VO2peak (maximum exercise capacity), E/e´ (diastolic function), NT-proBNP (N-terminal pro–brain-type natriuretic peptide). Significant values are in bold. §All tests were performed 2-sided. r* (Spearman's correlation coefficient).

		C16:1n7	C18:1n9	C20:1n9	C24:1n9
LDL-C	r*	**0.148**	-0.073	**-0.202**	-0.091
	p§	**0.006**	0.182	**<0.001**	0.093
non-HDL-C	r*	**0.257**	0.027	**-0.242**	**-0.18**
	p§	**<0.001**	0.616	**<0.001**	**0.001**
triglycerides	r*	**0.314**	**0.269**	**-0.128**	**-0.288**
	p§	**<0.001**	**<0.001**	**0.018**	**<0.001**
triglycerides-to-HDL-C ratio	r*	**0.305**	**0.299**	-0.101	**-0.257**
	p§	**<0.001**	**<0.001**	0.063	**<0.001**
ASAT	r*	**0.186**	0.094	-0.096	-0.084
	p§	**0.001**	0.083	0.077	0.12
ALAT	r*	**0.19**	0.054	**-0.156**	-0.037
	p§	**<0.001**	0.318	**0.004**	0.502
GGT	r*	**0.227**	0.102	**-0.114**	-0.081
	p§	**<0.001**	0.061	**0.035**	0.137
distance covered 6 MWT	r*	-0.098	**-0.161**	0.042	0.056
	p§	0.07	**0.003**	0.436	0.304
VO2peak	r*	-0.046	-0.036	0.06	**0.131**
	p§	0.396	0.506	0.266	**0.015**
E/e'	r*	-0.024	0.008	-0.069	0.009
	p§	0.658	0.889	0.202	0.863
NT-proBNP	r*	-0.11	0.008	0.099	0.073
	p§	0.042	0.881	0.068	0.182

Supplemental Table S3. Linear regression analyses of circulating MUFAs and patient characteristics **at baseline**. Abbreviations: LDL-C (low-density lipoprotein-cholesterol), non-HDL-C (non-high-density lipoprotein-cholesterol), HbA1c (hemoglobin A1c), ASAT (aspartate aminotransaminase), ALAT (alanine aminotransaminase), GGT (γ-glutamyltransferase), BMI (body-mass-index), 6 MWT (6 Minute Walk Test, i.e., sub-maximal exercise test), VO2peak (maximum exercise capacity), E/e′ (diastolic function), NT-proBNP (N-terminal pro–brain-type natriuretic peptide). Significant values are in bold. § All tests were performed 2-sided. β* (β-coefficient).

		**C16:1n7**	**C18:1n9**	**C20:1n9**	**C24:1n9**
LDL-C	β*	4.281	**-2.339**	**-232.088**	**-27.632**
	p§	0.383	**0.017**	**<0.001**	**0.007**
non-HDL-C	β*	**14.123**	-0.875	**-312.673**	**-49.467**
	p§	**0.010**	0.424	**<0.001**	**<0.001**
triglycerides	β*	**88.482**	**13.313**	**-307.830**	**-196.539**
	p§	**<0.001**	**<0.001**	**0.012**	**<0.001**
triglycerides-to-HDL-C ratio	β*	**2.171**	**0.391**	-6.356	**-4.552**
	p§	**<0.001**	**<0.001**	0.057	**<0.001**
HbA1c	β*	**0.291**	**0.065**	-0.565	**-0.464**
	p§	**0.001**	**<0.001**	0.532	**0.013**
ASAT	β*	**3.446**	0.363	-6.043	-3.983
	p§	**0.002**	0.101	0.589	0.086
ALAT	β*	**6.291**	**0.670**	-13.268	-5.389
	p§	**<0.001**	**0.043**	0.43	0.122
GGT	β*	**37.687**	**4.225**	-73.143	**-36.877**
	p§	**<0.001**	**<0.001**	0.115	**<0.001**
BMI	β*	**1.490**	**0.277**	-0.664	0.524
	p§	**<0.001**	**0.001**	0.875	0.549
waist circumference	β*	**3.540**	**0.855**	11.129	0.902
	p§	**0.002**	**<0.001**	0.329	0.704
waist-to-height ratio	β*	**0.023**	**0.005**	0.041	0.007
	p§	**0.001**	**0.001**	0.57	0.653
distance covered 6 MWT	β*	**-36.991**	**-8.566**	56.968	-6.198
	p§	**<0.001**	**<0.001**	0.537	0.746
VO2peak	β*	-0.453	**-0.167**	-5.405	-0.621
	p§	0.219	**0.022**	0.145	0.42
E/e'	β*	-0.292	-0.039	0.643	0.572
	p§	0.1	0.265	0.72	0.123
NT-proBNP	β*	**-0.104**	-0.001	**1.399**	**0.214**
	p§	**0.039**	0.887	**0.005**	**0.041**

Supplemental Table S4. Linear regression analyses of circulating MUFAs and patient characteristic **after 12 mFU**. Abbreviations: LDL-C (low-density lipoprotein-cholesterol), non-HDL-C (non-high-density lipoprotein-cholesterol), HbA1c (hemoglobin A1c), ASAT (aspartate aminotransaminase), ALAT (alanine aminotransaminase), GGT (γ-glutamyltransferase), BMI (body-mass-index), 6 MWT (6 Minute Walk Test, i.e., sub-maximal exercise test), VO2peak (maximum exercise capacity), E/e´ (diastolic function), NT-proBNP (N-terminal pro–brain-type natriuretic peptide). Significant values are in bold. § All tests were performed 2-sided. β* (β-coefficient).

		C16:1n7	C18:1n9	C20:1n9	C24:1n9
LDL-C	β*	2.840	**-2.606**	**-188.559**	**-24.558**
	p§	0.583	**0.011**	**<0.001**	**0.018**
non-HDL-C	β*	**15.434**	-0.533	**-253.709**	**-47.818**
	p§	**0.008**	0.645	**<0.001**	**<0.001**
triglycerides	β*	**110.818**	**16.699**	-244.930	**-170.159**
	p§	**<0.001**	**<0.001**	0.081	**<0.001**
triglycerides-to-HDL-C ratio	β*	**3.016**	**0.553**	-5.484	**-4.905**
	p§	**<0.001**	**<0.001**	0.25	**<0.001**
HbA1c	β*	0.207	**0.059**	-0.367	-0.292
	p§	0.073	**0.010**	0.746	0.213
ASAT	β*	**3.573**	0.224	**-21.804**	**-6.490**
	p§	**0.002**	0.320	**0.048**	**0.004**
ALAT	β*	**6.769**	0.387	**-38.837**	-5.608
	p§	**<0.001**	0.231	**0.014**	0.085
GGT	β*	**37.826**	**4.813**	-40.896	**-29.123**
	p§	**<0.001**	**<0.001**	0.364	**0.002**
BMI	β*	**1.775**	**0.315**	1.648	0.628
	p§	**<0.001**	**0.001**	0.717	0.501
waist circumference	β*	**4.627**	**1.038**	9.845	0.504
	p§	**<0.001**	**<0.001**	0.418	0.842
waist-to-height ratio	β*	**0.030**	**0.006**	-0.004	0.005
	p§	**<0.001**	**<0.001**	0.962	0.761
distance covered 6 MWT	β*	**-52.613**	**-8.582**	**248.995**	**47.358**
	p§	**<0.001**	**<0.001**	**0.026**	**0.041**
VO2peak	β*	**-1.186**	**-0.256**	1.208	1.703
	p§	**0.034**	**0.021**	0.819	0.119
E/e'	β*	-0.308	-0.025	-0.989	0.236
	p§	0.137	0.537	0.624	0.569
NT-proBNP	β*	-0.055	0.001	2.156	0.202
	p§	0.661	0.971	0.072	0.412

In figures 2 and 3, the r and p values should be identified in the figure or mentioned in the legend.

Thank you, we have accordingly added the r and p values in the figure legends of figures 2 and 3.

Some abbreviations are presented without previous definition and others are defined more than one time.

Thank you for bringing this to our attention. We have now defined all abbreviations upon first mentioning them and we have omitted all abbreviations that were defined twice.

Reviewer #2:

Comments and Suggestions for Authors: None

We would like to thank the reviewer for his time and for reviewing our work.
